# Supplementary figures and images for: Genetic variability of bioactive compounds and selection for nutraceutical quality in kola [Cola nitida (Vent) Schott. and Endl.]
Source: PLoS One. 2020 Dec 3;15(12):e0242972. doi: 10.1371/journal.pone.0242972 (PMC7714174; doi:10.1371/journal.pone.0242972)

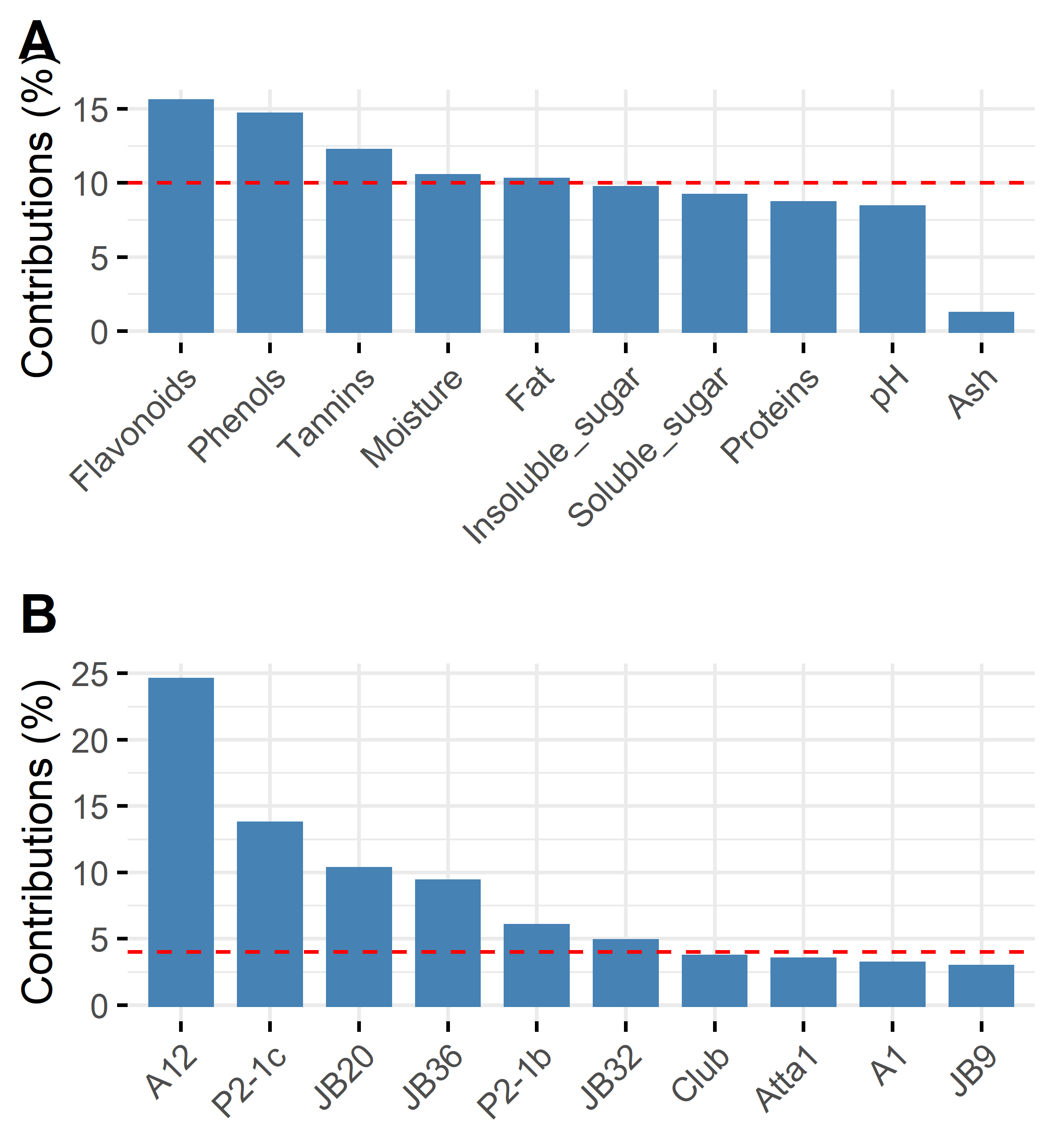

Supplement: S1 Fig — Variables and individuals cut by the red dashed lines are significantly represented on the the first two principal components. (TIF) [file pone.0242972.s001.tif]
