# Supplementary material for: Genetic variability of bioactive compounds and selection for nutraceutical quality in kola [Cola nitida (Vent) Schott. and Endl.]
Source: PLoS One. 2020 Dec 3;15(12):e0242972. doi: 10.1371/journal.pone.0242972 (PMC7714174; doi:10.1371/journal.pone.0242972)
Supplement: Attachment — Submitted filename: Response to reviewers.docx [file pone.0242972.s003.docx]

**Response to Comment 1.** We are particularly shocked about the idea that a paper is rejected because someone thought that the crop the manuscript targeted is a minor crop inAfrica. Sorry, but **Minor crops in Africa** also **or should** also **matter in Science.** In fact, PLoS ONE clearly indicates that: “**it evaluates submitted manuscripts on the basis of methodological rigor and high ethical standards, regardless of perceived novelty**”. Prof Preasanda K. Subuthi did not point out any methodological pitfall in this manuscript. We strongly believe that this decision needs to be reviewed since we are confident about the methodological rigour we used in the manuscript. We would be glad if the manuscript could be reassessed by another editor who truly knows the decision’s rules of the respected PLoS One Journal.

**Editor’s comment 2. The literature presented in the manuscript omitted several studies in this crop**

**Response to Comment 2.** We would like to recall that this manuscript is not a review, so we only used literatures relevant to the specific aspects tackled in the manuscript. In addition, the editor comment seemed vague and did not point out specifically what he would like to see in the manuscript’s background. We would be happier if he indicated what he thought we did not take into account. We are confident that we used the last literature on the species Genetics and breeding.

**Editor’s comment 3.** This study is too preliminary and does not advance science to the next level.

**Response to Comment 3.** Our study is the first in its kind to screen variability of bioactive compounds in *Cola nitida*. It offers first-order decision making information (e.g. heritability, genetic gain) for advancing elite cultivar development in this minor crop. It is well known that most of the minor crops are poorly documented from many points of view, and for such crops everything is to be built, starting from what those working on major crops see as preliminary. The fact that Prof Subuthi working on rice should not let think that others’ contribution is not valuable.

In summary, we did not see this decision of Prof Subithi objective. Is our methodology sound? Do we adhere to PLoS ONE ethics requirement in the manuscript? Do our results support our conclusion? Are we transparent with our data? Those were the points we expected the decision to be based on.

To Prof Subithi, we would like to say that he can stick to his rice, but we, we are writing a history, that of the African Orphan Crop. African Orphan Crop matter.
