# Supplementary material for: Genetic variability of bioactive compounds and selection for nutraceutical quality in kola [Cola nitida (Vent) Schott. and Endl.]
Source: PLoS One. 2020 Dec 3;15(12):e0242972. doi: 10.1371/journal.pone.0242972 (PMC7714174; doi:10.1371/journal.pone.0242972)
Supplement: Attachment — Submitted filename: Response to the reviewers.docx [file pone.0242972.s004.docx]

Response to the reviewers

While the study provides a baseline for evaluating kola genotypes for breeding purposes and will be of interest to a range of scientists and breeders working on orphan crops, the manuscript needs to be extensively corrected for content (specifically materials and methods), grammar, spelling, syntax and formatting before it can be considered for publication in PloSONE.

Response: We wish to thank the editor for his positive comments and suggestions to fine tune the manuscript. The corrections suggested were accepted in good faith. Corrections have been carried out on the materials and methods, grammar, spelling, syntax and formatting as suggested. These corrections could be seen in the manuscript with the track changes. Thank you.

1. Information on the experimental materials used in the study needs to be clarified. In page 5, line 92, the authors mentioned using 25 clones of C. nitida. Clones are individuals having similar genetic constitution. For a study aimed at looking at genetic variability in any trait, the use of clones does not make any sense since each of these individuals will have the same genotype, ergo the same phenotype. Even if variation for a specific trait is observed, the variation would not be significant. But looking at Table 1, significant variation in pod yield and nut weight and color among the clones have been reported. While yield and weight are quantitative traits that are affected by the environment, the data presented in Table 1 does not conform to the expected intra-clonal variation. To make matters more complicated, in page 8, line 116, the authors mentioned ‘grafting’ in relation to the 25 genotypes used in the study. It is unclear if the materials used in the study were scions from a single plant (clones) grafted to 25 different rootstock genotypes or 25 different scions grafted to the same rootstock genotype? This is important especially because one of the objectives of the study is identifying genotypes that can be used in breeding. If the materials used in the study are indeed grafts, the interaction between scion and grafts might have some effect in the traits examined and this should be determined prior to recommending genotypes that can be used as donors in breeding.

**Response**

The 25 clones means 25 genotypes of kola. We refer to them as clones because they were vegetatively propagated through grafting and planted as a research trial. The 25 genotypes do not have the same genetic constitution and their phenotypes are not the same. The cuttings of the 25 genotypes were grafted on a rootstock of one genotype or of same genetic background. Therefore, there was no rootstock and scion interaction. To make it clear to our readers, the term “clones” has been changed to “genotypes” and the paragraphs rephrased to make issues clear to our readers. The changes can be seen clearly in the manuscript with track changes. Thank you.

2. Page 4 line 67-73. The first and last sentence of this paragraph are contradictory.

**Response:** Comment has been considered in revising the manuscript. The sentence has been rephrased to take out the contradiction.

3. Details about the methodology used for nitrogen, protein, carbohydrate and phenolic analysis should be provided. The readers should not have to go back to the cited papers for the full method.

**Response:** Details of the methodology for nitrogen, protein, carbohydrate and phenolic analysis has been provided in revising the manuscript. Thank you.

4. The manuscript needs to be checked for several grammatical, typographical and spelling errors, formatting, as well as syntax to improve the overall readability of the paper.

**Response:** This comment has been considered in revising the manuscript. The corrections on grammar, typographical and spelling errors, formatting and syntax was carried out throughout the manuscript.

Examples:

a. Page 3, line 45: Cola acuminate should be changed to C. acuminate

Response: The correct one is *Cola acuminata.*

b. Page 3, lines 49-50: This sentence is missing a period.

Response:

c. Page 3, line 51: “The kola industry offers” not “offer”

Response: Thank you for the correction. The correction has been implemented.

d. Page 3, line 59-60: Sentence can be paraphrased as “Bioactive compounds (phytonutrients) such as carotenoids and phenolic acids are health-promoting compounds that act against cardiovascular diseases and various types of cancer.”

Response: Thank you for the correction. The correction has been implemented.

e. Page 4, line 74: ‘bioactive compounds’ should be changed to ‘bioactive compound’

Response: Thank you for the correction. The correction has been implemented.

f. Page 4, line 74: wrong spelling of ‘content’ and ‘greatly’

**Response:** Thank you so much for identifying the spelling mistakes. Corrections has been implemented. They were rightly spelled.

g. Page 4, line 74: ‘ …contents of fruits are’ not ‘….is’

Response: Thank you for the correction. Correction has been implemented.

h. Page 4, line 80-82: Sentence needs to be corrected. Wrong usage of grammar and sentence correction.

Response: Thank you for pointing this out. The sentence has been rephrased and grammar corrected. This can be clearly seen in the manuscript with track changes.

i.Page 5, line 99: Use the symbol for ‘degrees’ instead of superscripting the number ‘zero’

Response: Thank you for the correction. The correction has been implemented. The symbol of “degrees” was used.

j. Page 5, line 102: Remove comma after April.

Response: Thank you for the correction. The correction has been carried out.

k. Page 8, line 116: The phrase “the grafted materials the 25 kola genotypes….’ Is unclear. Please paraphrase.

Response: Thank you for the correction. Those phrases has been paraphrased to make them clearer.

l. Page 11, line 169: Subheading title is unclear. Please paraphrase.

Response: Thank you for the correction. The subheading has been paraphrased to make it clear. The corrections can be clearly seen in the manuscript with track changes.

m. Page 11, line 171: wrong spelling of ‘between’

Response: Thank you for the correction. The Correction has been implemented.

n. Page 11, lines 174, 176 and 177: What does the ‘()’ after the stat test mean?

Response: It is a script language which translates the way functions are written in R.

o. Page 16, lines 259-261: the five variable mentioned need not be in bold. Also, flavonoids and phenols should not be capitalized.

Response: Thank you for the correction. The corrections were considered in revising the manuscript.

p. Page 16, line 264: change ‘………flavonoids and phenols contents’ to ‘…..flavonoid and phenol contents’.

Response: Thank you for the correction. The correction has been carried out.

q. Page 17 line 271: Remove ‘an’ in ‘Breeding fruits with an enhanced nutritional…’

Response: The correction was considered in revising the manuscript. Thank you.

r. Page 17, line 274: change has to have

Response: The correction was considered in revising the manuscript. Thank you.

s. Page 17, line 283: Put a space between ‘…nutraceutical contents’ and the citation [48]

Response: The correction was considered in revising the manuscript. Thank you.

t. Page 21, line 322: No space after the sentence and the period.

Response: The correction was considered in revising the manuscript. Thank you.

u. Page 21, line 323: Add a space between ‘in’ and ‘a’.

Response: The correction was considered in revising the manuscript. Thank you.

v. The manner by which P values were written were not consistent across the manuscript.

Response: The correction was considered in revising the manuscript. The manner in which P values are written is now uniform across the manuscript. Thank you.

w. Tables should be re-formatted according to PLoSONE specifications.

Response: Comment was considered in revising the manuscript. Tables has been formatted according to PLoSONE specifications.

x. The references should be checked for formatting and typographical and spelling errors.

Response: Comment was considered in revising the manuscript. All references were listed and well formatted. Typographical and spelling errors has been corrected.

The whole manuscript was revised and corrections were carried out. Corrections can be seen in the manuscript with track changes.
